# Evaluation of Microbial Enzymes in Normal and Abnormal Cervicovaginal Fluids of Cervical Dysplasia: A Case Control Study

**DOI:** 10.1155/2014/716346

**Published:** 2014-05-22

**Authors:** Subramanyam Dasari, Wudayagiri Rajendra, Lokanatha Valluru

**Affiliations:** ^1^Department of Biotechnology, Dravidian University, Kuppam 517426, India; ^2^Department of Zoology, Sri Venkateswara University, Tirupati 517502, India

## Abstract

The aim of the present study was to evaluate the role of microbial enzymes in normal and abnormal cervicovaginal fluids of cervical dysplasia. The cervicovaginal infections were evaluated through the estimation of microbial enzymes in patients with and without abnormal cervical cytology like bacterial and fungal infections. The patients were categorized based on infection caused by organism and stages of dysplasia. The pH, Whiff test, and Pap smear tests were conducted for normal and abnormal cervical swabs based on standard protocols. Microbial enzymes include mucinase, sialidases, and proteases of the cervical swabs and are estimated according to standard methods. The results of abnormal cervical cytological smears showed increased pH and the presence of amines with different levels of Pap smear test. Increased levels of microbial enzymes were observed in patients with abnormal cytology than normal cytology. Three microbial enzymes mucinase, sialidase, and protease were significantly (*P* < 0.01) more elevated in patients with bacterial infections (8.97 ± 0.64, 10.39 ± 0.28, 8.12 ± 0.64) than without dysplasia (2.02 ± 0.8, 1.98 ± 0.3, 1.96 ± 0.8). The results reinforce that the microbial infection seems to be more prone to cervical dysplasia and may act as risk-factor for the development of cervical cancer along with HPV infection.

## 1. Introduction

The human papillomavirus (HPV) is one of the most common sexually transmitted pathogens and is strongly associated with women health burden like uterine cervical cancer. At least 50% of men and women acquire genital HPV infection during their lifetime [1]. HPV is not only associated with cases of cervical cancer but also causes vulvar, vaginal [2, 3], and genital warts and respiratory papillomatosis [4]. Most of the women individuals are not aware of the fact that they are infected with HPV because of its subclinical or asymptomatic appearance and thus, the virus can be spread easily and unknowingly during sexual intercourses [5]. It was established that HPV infection alone may not be enough to promote cervical carcinogenesis and some other cofactors such as smoking, immunosuppression, oral contraceptives, and vitamin deficiency, and other sexually transmitted diseases such as bacterial vaginosis (BV) (*Chlamydia trachomatis*) and protozoan infections (*Trichomonas vaginalis*) are also involved in the development of cervical cancer [6, 7]. Many pathogenic agents have been studied as risk factors which interact with HPV in the development of precancerous and cancerous lesions of the cervix. The abnormal vaginal flora can produce carcinogenic substances (nitrosamines) or other metabolites which could be directly or indirectly intricate in the development of cervical lesions by increasing the susceptibility of the inflamed epithelium [8].

Under the normal physiological conditions, healthy vagina contains lactobacilli that function mutually with vaginal epithelium, colonizing and making it resistant to other pathogenic microorganisms, and prevent ascendant or systemic infections [9]. The infection of high risk human papilloma viruses (HR-HPV) to the susceptible epithelial cells of uterine cervix leads to the common treatable vaginal infections that disrupt the intricately balanced vaginal ecosystem. Earlier reports have suggested an association between abnormal vaginal discharge and cervical intraepithelial neoplasia (CIN) followed by cervical cancer [10].

Bacterial vaginosis (BV) and other infections are associated with high levels of anaerobic microorganisms and their by-products (enzymes), which can damage the vaginal epithelium, degrade cervical mucus cells, and cleave immunoglobulin-A (Ig-A) [11]. A large body of evidence suggested that an important association between HR-HPV and alterations in the vaginal microbiome leads to the development of uterine cervical cancer [12]. BV and trichomoniasis are characterized, with vulvovaginal candidiasis under the slight misnomer of vaginitis.* Candida albicans* is the most predominant species in the majority cases of asymptomatic colonization and vulvovaginal candidiasis. However, certain species of* Candida* are more pathogenic and induces hyphal, pseudohyphal formation, which enhances the proteolytic activity and antigen modulation. These characteristics enable* Candida* to penetrate the mucosal surface and induce mucosal swelling, erythema, and exfoliation of cells [13].

In addition, some previous studies have stated that cervical cytologic abnormalities occur more frequently in women who have abnormal vaginal microbiota than in women without this condition. The women who carry the abnormal microbiota are more prone to acquiring cervical cytologic abnormalities than women without microbial infections [12].

Under this backdrop, the present study investigates role of microbial enzymes as risk factors in the development cervical cancer. Therefore, the levels of microbial enzymes were estimated in normal and abnormal cervicovaginal fluids of cervical dysplasia that degrade the mucus membrane and epithelial cells of uterine cervix.

## 2. Materials and Methods

### 2.1. Sample Collection

Cervicovaginal smear samples were collected from pathology laboratory, Department of Obstetrics and Gynecology, Government Maternity Hospital, Tirupati, and PES Institute of Medical Sciences and Research, Kuppam, Andhra Pradesh, India, who (*N* = 109) had abnormal smears or cervicograms. Ten smears were also collected from healthy women who do not have any symptoms or vaginal diseases. Care was taken to avoid contamination of cervical smears and the swabs were immediately transferred into −20°C for further analysis.

#### 2.1.1. Ethical Approval

The objectives and methods of the study were clearly explained to the participants. Written consent from participants as well as their guardians was obtained before collection of data. The present work was approved by the Institutional Ethical Committee (IEC), Sri Venkateswara (S.V) Medical College, Tirupati, Andhra Pradesh, India, (Rc. no:A1/SPL/GMH/TPT/2012, Dt: 12.01.12) along with the patient consent forms.

### 2.2. pH and Amines (Whiff) Test

Vaginal discharge and odour are frequent gynaecological complaints that result in women seeking medical care. A portion of the undiluted vaginal material and one drop of a saline suspension was applied on the surface of a clean glass slide. One drop of 10% potassium hydroxide (KOH) was added to the vaginal sample. The presence of volatile amines which have a fishy odour [14] gives positive result.

### 2.3. Pap Smear Tests

A small amount of undiluted smear was fixated with cytological fixative and colored reagent by standardized method according to Papanicolaou, [15] which is modified with automated Papanicolaou smear analysis by Ku, [16]. Papanikolau test results were presented in the class system for reporting cervical smear.

### 2.4. Detection of Microbial Infection

The presence of bacteria and fungi was evaluated microscopically in samples collected from the posterior vaginal fornix. Microbial infection was diagnosed on the basis of clinical and microscopic findings.

### 2.5. Determination of Microbial Enzymes

#### 2.5.1. Mucinase Activity

Cervical swabs were mixed with 0.01 M sodium phosphate (pH: 7.5) at a ratio of 1 : 100 (Wt/Wt) and allowed to stand for 20 minutes to promote the softening and homogenate the cervical smears.

Cervical homogenate (0.9 mL) was allowed to incubate at 30°C for 2 min and 0.1 mL of 0.5% mucin (Sigma Aldrich) was added. The reaction mixture was incubated at 30°C for 25 minutes and then placed into boiling water for 3 minutes to stop the enzymatic reaction. The released reducing sugars were measured by DNS method at 520 nm [17]. Mucinase specific activity and total output were expressed as micromoles glucose equivalents/(minute-milligrams).

#### 2.5.2. Determination of Protease Activity

The protease enzyme activity was measured in duplicate by measuring the release of trichloroacetic-acid soluble peptides from 0.2% (w/v) azocasein in 50 mM HEPES/NaOH buffer (pH 7.5) at 50°C for 10 min. The reaction was terminated by the addition of 0.5 mL of 15% trichloroacetic acid and then centrifuged at 10,000 rpm for 10 min. One unit (U) enzyme activity was defined as the amount of enzyme required to produce an increase in absorbance at 420 nm equal to 1.0 in 60 min under the assay conditions [18].

#### 2.5.3. Sialidase Activity

The glycoprotein sialidase activity was measured quantitatively by the method of Howe et al. [19] using human alpha-1 acid glycoprotein (AGP). Cervical smear and substrate (AGP) were incubated at 37°C for 20–30 min for the hydrolysis of sialic acid present in substrate. After incubation, the free sialic acid was measured using the thiobarbituric acid assay. This assay measures bacterial enzyme activities associated with the process of sialic acid removal from sialoglycoproteins, intricate in mucin degradation.

## 3. Results

**Figure 1:**
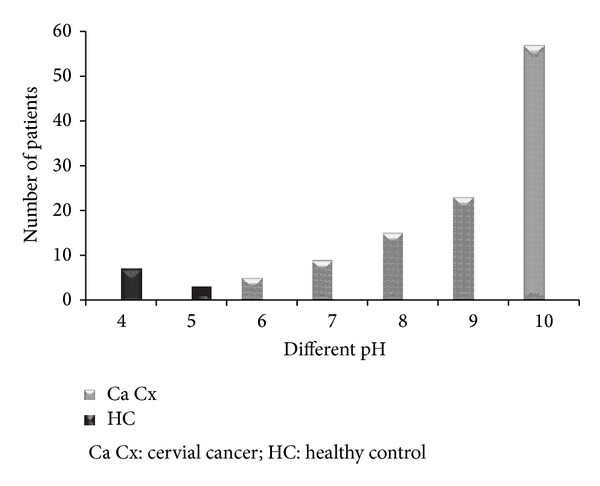
pH of the cervicovaginal smears of healthy and cervical cancer patients. Healthy cases show very low pH (pH 4 and 5) and cancer patients show increased pH (6 to <11).

All tested patients show (Figure 1) the increased pH ranging from 6 to 10 which is more than the healthy vaginal pH (<4.5). Of the tested 109 patients, 89 patients (81%) give volatile amines which have a fishy odour and 20 cases (18.34%) did not give any fishy odour.

### 3.1. Pap Smear Test for Abnormal Cervical Vaginal Infections

**Figure 2:**
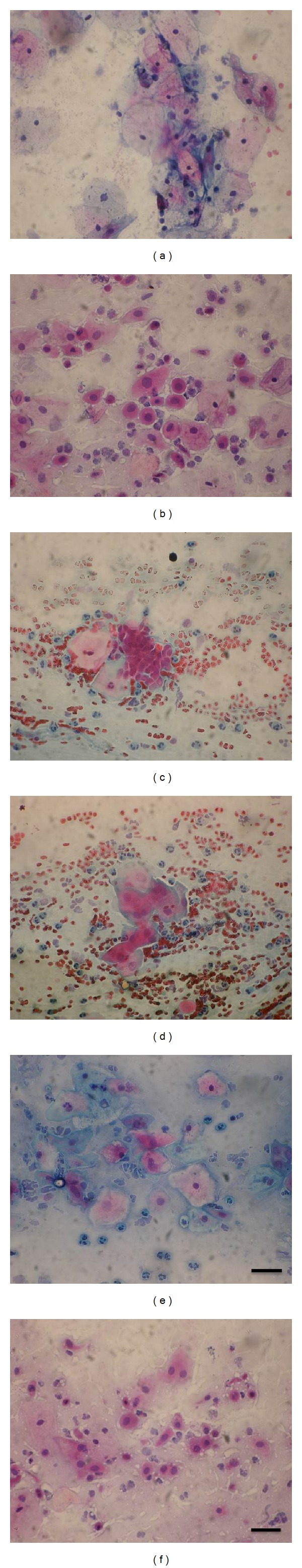
Pap smear test of the cervicovaginal smears showing different stages of dysplasia in 40x magnification. The figure shows the normal cervical cells, atypical squamous cells with irregular nuclei, with hyper chromatin and pleomorphic nature. (a) Normal cytology of cervical swabs; (b) ASCUS: atypical squamous cells of undetermined significance; (c) ASC-H: atypical squamous cells, cannot exclude high-grade squamous intraepithelial lesion with hyper chromatin; (d) ASC-H: atypical squamous cells, cannot exclude high-grade squamous intraepithelial lesion with pleomorphism; (e) L-SIL: low-grade squamous intraepithelial lesion; (f) H-SIL: high-grade squamous intraepithelial lesion. Lens 40x. Bars = 10 *μ*m.

In the present investigation, 109 Pap smears were screened and categorized into 5 subgroups of patients with abnormal vaginal cytology: (1) atypical squamous cells of undetermined significance (ASCUS); (2) atypical squamous cells, cannot exclude high-grade squamous intraepithelial lesion (ASC-H); (3) low-grade squamous intraepithelial lesion (L-SIL); (4) high-grade squamous lesion (H-SIL); (5) negative for pap smear test. Of the tested 109 cervical smears, 14 (7.3%) smears belong to ASCUS with enlarged cell nucleus and the cytoplasm (Figure 2(a)), increased nuclear (NC) ratio, and irregular nuclear membrane. In 18 cases (16%), observed as those atypical squamous cells, not excluding high-grade squamous intraepithelial lesions (ASC-H) which are characterized by the presence of hyperchromatin, pleomorphic nature of cells (Figure 2(b)) and their exact nature are uncertain. There is no evidence of cancer in this stage. In 19 cases (17%) of Pap smear, data indicated that cells with abnormal changes are consistent with HPV (human papilloma virus) infection. These types of low score or undeveloped abnormal cells are not enough to justify a “definite” diagnosis. This is an early step in the development of cervical cancer according to the CIN classification. 50 (45%) of the tested smears were identified as H-SIL, with features suspicious for invasion (if invasion is suspected) and it is characterized by the abnormal squamous cells from moderately to severely infected cells. Eight cases were observed as negative for Pap smear test.

In the present study, the cytopathological observations of cervical cancer patients were categorized based on types of microbial infection and stages of dysplasia (cervical intraepithelial neoplasia-CIN). Microbial infections are bacterial (*N* = 38), fungi alone (*N* = 31), and fungi with candida infection (*N* = 20) along with negative samples without any abnormal cervical cytology (*N* = 20). Cervical dysplasia is divided into cervical intraepithelial neoplasia I (*N* = 20), cervical intraepithelial neoplasia II (*N* = 21), cervical intraepithelial neoplasia III (*N* = 18), cervical carcinoma* in situ* (*N* = 25), and intraepithelial cervical carcinoma (*N* = 25) cases. All the characteristics of the patients were shown in Table 1.

### 3.2. Determination of Microbial Enzymes

The mean values of mucinase were estimated in all the four groups of microbial infections, in which the mucinase enzyme was significantly increased in bacterial infections (8.97 ± 0.64 ng/mL) when compared to the remaining infections and also healthy cases (0.92 ± 0.05 ng/mL). The enzymes sialidase and protease were also significantly elevated in the group of bacterial infections (10.39 ± 0.28, 8.12 ± 0.64 ng/mL) when compared to the remaining cases and also healthy cases (0.91 ± 0.06, 0.47 ± 0.02 ng/mL).

Based on the cervical intraepithelial neoplasia (CIN) categories, the microbial enzymes, mucinase, sialidase, and protease were significantly (*P* < 0.01) elevated in intraepithelial cervical carcinoma (ICC) (8.42 ± 0.58, 10.28 ± 0.34, 7.68 ± 0.91 ng/mL) when compared to the remaining CIN types.

## 4. Discussion

Microbial infection is the most frequent cause of bacterial vaginitis and is characterized by increase in growth of anaerobic bacteria and also an unbalanced microbial ecosystem in the vagina [6]. Vardar et al. [20] reported that microbial infection is a cause of abnormal cytology in cervical cancer and also reported that there are high sensitivity and specificity values of Gram stain in diagnosis of bacterial vaginosis (BV), besides Amsel's criteria [21].

The clinicopathologic condition characterized by redness in the vaginal wall, bad-odoured discharge, and the presence of clue cells in cervicovaginal specimens, which results from the transformation of the acidic vaginal pH to an alkaline pH via metabolic activity of bacterial compounds like nitrosamines [22]. The products of anaerobic infection responsible for fishy smell-putrescine, cadaverine, diethylamine, and succinate are increased in the vaginal washings of women with bacterial infection and the lactate/succinate ratio has been used as a biochemical marker for bacterial infection [23]. It was also reported that microbial infection causes premature rupture of membranes, preterm delivery, and endometritis. BV has the ability to develop possible carcinogenic effects due to abnormal vaginal cytology and mosaic colposcopic pattern in the cervix [24, 25]. It was well established that various methods have been recommended for the evaluation of microbial infection or evaluate the risk factor by cytological studies.

In the present study, different types of microbial population were observed in abnormal cervicovaginal swabs, and most of these bacteria are anaerobic bacteria like* Salmonella*,* Gardnerella*,* Chlamydia*, and* Neisseria*. The replacement of the normal hydrogen peroxide-producing* Lactobacillus* with anaerobic bacteria leads to complications associated with many obstetric and gynaecological disorders including preterm labor and delivery, postcesarean endometritis, chorioamnionitis, pelvic inflammatory disease (PID), and a possible connection with abnormal cervical cytology and finally leads to CIN [26, 27].

In the present investigation, the statistical (Duncan's multiple range test, ANOVA) data of microbial enzymes are shown in Table 1, which indicated that the three enzymes were more augmented in patients with abnormal cervicovaginal discharges than the normal discharges. Of the tested microbial infections, the bacterial infections show significant (*P* < 0.01) more elevated levels of enzymes mucinase, sialidase, and protease than the remaining infections. The increased production of enzymes mucinase, sialidases, and protease is due to the increased ratio of pathogenic anaerobic bacteria. Larsen and Monif [28] demonstrated that the ratio of (10 : 1) anaerobic bacteria outnumbered aerobic bacteria in women of reproductive age. This ratio clearly reflects a dynamic colonization process and increased production of enzymes. The activity of these enzymes plays a main role in the degradation of mucus membranes of the cervix, which facilitates the entry of HPV and then leads to the development of cervical intraepithelial neoplasia (CIN). Hence, the levels of enzymes were more elevated at intraepithelial cervical carcinoma (ICC) than the CIN.

The possible relationship between bacterial infection and cervical intraepithelial lesions has been proposed since the 1970s. Platz-Christensen et al. [27] found that the presence of grades 1, 2, and 3 CIN in 5% of women who also presented BV and only 1.4% of women without BV. Eltabbakh et al. [29] found that 50% of women with cervical abnormalities at Pap smear test had a cervicovaginal infection, 28% of which were BV. Other bacteria,* Chlamydia trachomatis* (Ct), infection acts as a risk factor in the development of cervical lesions. Strong epidemiological evidence suggested that combined infection with HPV and Ct plays a lynchpin in the etiology of intraepithelial lesion of the uterine cervix and leads to the subsequent development of invasive cervical neoplasia when associated with other factors, such as smoking and sexual promiscuity [7]. The association of protozoan infections (*Trichomonas vaginalis* (Tv)) with cervical lesion has been studied from the 1950s. Zhang and Begg [30] reported a double risk of developing intraepithelial lesions in the presence of Tv.

## 5. Conclusion

In conclusion, the abnormal cervicovaginal smears show elevated pH and the presence of fishy odour is more frequent in cervical swabs with abnormal cytology. The elevated levels of microbial enzymes were observed in patients with abnormal cytological dysplasia than the normal dysplasia indicated that microbial enzymes act as cofactors for cervical cancer. Hence, the patients with abnormal cervicovaginal fluids of cervical dysplasia are more prone to acquire cervical cancer than the normal dysplasia patients.

## Figures and Tables

**Table 1:** Microbial enzyme (mucinase, sialidase, and protease) in cervical swabs of both healthy, cervicovaginal infections and different stages of dysplasia conditions.

S. no.	Character	Mean (SD) ng/mL			
		*N *	Mucinase	Sialidase	Protease
1	Healthy controls	10	0.92 ± 0.05	0.91 ± 0.06	0.47 ± 0.02

	Based on microbial infections				
2	Bacterial infections	38	8.97 ± 0.64*	10.39 ± 0.28*	8.12 ± 0.64*
	Fungal infections	31	4.96 ± 0.24**	8.52 ± 0.28**	6.35 ± 0.53**
	Fungi + *Candida *	20	3.75 ± 0.40^$^	8.62 ± 0.85^$^	5.37 ± 0.44^$^
	Negative	20	2.02 ± 0.8	1.98 ± 0.3	1.96 ± 0.8

	Based on CIN				
3	CIN I	20	3.05 ± 0.26	5.98 ± 0.46	3.57 ± 0.56
	CIN II	21	3.55 ± 1.13	4.14 ± 0.69	4.56 ± 0.83
	CIN III	18	6.80 ± 0.77**	9.05 ± 0.98**	6.59 ± 0.81**
	CIS	25	5.96 ± 0.62^$^	9.77 ± 0.52**	6.98 ± 0.93**
	ICC	25	8.42 ± 0.58*	10.28 ± 0.84*	7.68 ± 0.91*

CIN: cervical intraepithelial neoplasia; CIS: carcinoma *in situ*; ICC: intraepithelial cervical carcinoma. Comparison between normal (healthy) and abnormal cervical cytology like bacterial, fungal, fungi + *Candida* infections. *Significant with *P* < 0.01; **P < 0.05; ^$^ *P* < 0.25.

## References

[1] Paavonen J (2007). Human papillomavirus infection and the development of cervical cancer and related genital neoplasias. *International Journal of Infectious Diseases*.

[2] Daling JR, Madeleine MM, Johnson LG (2005). Penile cancer: Importance of circumcision, human papillomavirus and smoking in in situ and invasive disease. *International Journal of Cancer*.

[3] Daling JR, Madeleine MM, Schwartz SM (2002). A population-based study of squamous cell vaginal cancer: HPV and cofactors. *Gynecologic Oncology*.

[4] Hatch KD, Berek JS, Berek JS (2002). Intraepithelial diseases of the cervix, vagina and vulva. *Novaks Gynecology*.

[5] Moscicki AB (2005). Impact of HPV infection in adolescent populations. *Journal of Adolescent Health*.

[6] Nam KH, Kim YT, Kim SR (2009). Association between bacterial vaginosis and cervical intraepithelial neoplasia. *Journal of Gynecologic Oncology*.

[7] Castellsagué X, Bosch FX, Muñoz N (2002). Environmental co-factors in HPV carcinogenesis. *Virus Research*.

[8] Bornstein J, Rahat MA, Abramovici H (1995). Etiology of cervical cancer: current concepts. *Obstetrical and Gynecological Survey*.

[9] Zoran J, Zoran PT, Miroslav F, Jasmina P, Aleksandra P, Predrag V (2011). *Frequency of cervical intraepithelial neoplasia and carcinomas in women with and without bacterial vaginosis acta medica medianae*.

[10] Venegas G, Boggiano G, Castro E (2011). Prevalence of bacterial vaginosis in Chilean sex workers. *Revista Panamericana de Salud Publica*.

[11] Li C, Wu M, Wang J (2010). A population-based study on the risks of cervical lesion and human papillomavirus infection among women in Beijing, People’s Republic of China. *Cancer Epidemiology Biomarkers and Prevention*.

[12] Dai Q, Hu L, Jiang Y (2010). An epidemiological survey of bacterial vaginosis, vulvovaginal candidiasis and trichomoniasis in the Tibetan area of Sichuan Province, China. *European Journal of Obstetrics Gynecology and Reproductive Biology*.

[13] Ganguly S, Mitchell AP (2011). Mucosal biofilms of Candida albicans. *Current Opinion in Microbiology*.

[14] Plourd DM (1997). Practical guide to diagnosing and treating vaginitis. *Medscape Womens Health*.

[15] Papanicolaou GN (1949). A survey of the actualities and potentialities of exfoliative cytology in cancer diagnosis. *Annals of internal medicine*.

[16] Ku NN (1999). Automated Papanicolaou smear analysis as a screening tool for female lower genital tract malignancies. *Current Opinion in Obstetrics and Gynecology*.

[17] Shiau SY, Chang GW (1983). Effects of dietary fiber on fecal mucinase and-glucuronidase activity in rats. *Journal of Nutrition*.

[18] Janssen PH, Peek K, Morgan HW (1994). Effect of culture conditions on the production of an extracellular proteinase by Thermus sp. Rt41A. *Applied Microbiology and Biotechnology*.

[19] Howe L, Wiggins R, Soothill PW, Millar MR, Horner PJ, Corfield AP (1999). Mucinase and sialidase activity of the vaginal microflora: Implications for the pathogenesis of preterm labour. *International Journal of STD and AIDS*.

[20] Vardar E, Maral I, Inal M, Özgüder Ö, Tasli F, Postaci H (2002). Comparison of Gram stain and Pap smear procedures in the diagnosis of bacterial vaginosis. *Infectious Disease in Obstetrics and Gynecology*.

[21] Lamont RF, Hudson EA, Hay PE (1999). A comparison of the use of Papanicolaou-stained cervical cytological smears with Gram-stained vaginal smears for the diagnosis of bacterial vaginosis in early pregnancy. *International Journal of STD and AIDS*.

[22] Priestley CJ, Kinghorn GR (1996). Bacterial vaginosis. *British Journal of Clinical Practice*.

[23] Piot P, van Dyck E (1983). Isolation and identification of Gardnerella vaginalis. *Scandinavian Journal of Infectious Diseases*.

[24] Hauth JC, Goldenberg RL, Andrews WW, Dubard MB, Copper RL (1995). Reduced incidence of preterm delivery with metronidazole and erythromycin in women with bacterial vaginosis. *The New England Journal of Medicine*.

[25] Hillier SL, Nugent RP, Eschenbach DA (1995). Association between bacterial vaginosis and preterm delivery of a low-birth-weight infant. *The New England Journal of Medicine*.

[26] Morris M, Nicoll A, Simms I, Wilson J, Catchpole M (2001). Bacterial vaginosis: a public health review. *British Journal of Obstetrics and Gynaecology*.

[27] Platz-Christensen JJ, Sundstrom E, Larsson P-G (1994). Bacterial vaginosis and cervical intraepithelial neoplasia. *Acta Obstetricia et Gynecologica Scandinavica*.

[28] Larsen B, Monif GR (2001). Understanding the bacterial flora of the female genital tract. *Clinical Infectious Diseases*.

[29] Eltabbakh GH, Eltabbakh GD, Broekhuizen FF, Griner BT (1995). Value of wet mount and cervical cultures at the time of cervical cytology in asymptomatic women. *Obstetrics and Gynecology*.

[30] Zhang ZF, Begg CB (1994). Is Trichomonas vaginalis a cause of cervical neoplasia? Results from a combined analysis of 24 studies. *International Journal of Epidemiology*.

